# Enlarged Prostate

**Published:** 1902-04-12

**Authors:** 


### ENLARGED PROSTATE.

With the object of ascertaining what it really is that is removed in the operation of prostatectomy, in which, as is claimed by some, the whole prostate is enucleated "in its capsule," Mr. Cuthbert Wallace [1] has made a careful examination of many sections of various enlarged prostates. The points which he endeavours to determine are the nature of the pathological alteration in the organ, the nature and origin of the "capsule," and the relation which this bears to the normal sheath. To do this numerous specimens have been examined, and in all the same changes, though in different degree, have been found. It would seem that the first pathological change is an unequal growth of the glandular elements. The more rapidly-growing areas increase at the expense of the more slow-growing ones, which are compressed and stretched over the surface of their quickly-growing neighbour. By this process a capsule is formed, ill-defined at first, but later becoming very distinct. The adenomatous mass can now be easily enucleated, and not only presents a smooth surface, but also leaves behind a smooth-lined cavity. These adenomata are often, indeed usually, compound, and may occupy the whole space within the "capsule." What then is this capsule? In the normal state there certainly is no capsule comparable to that of the kidney, for the fibrous covering is intimately combined with the organ, and except for a small area on the rectal surface any attempt at separating it simply leads to tearing the prostatic tissue. There can be no doubt, Mr. Wallace says, that the capsule met with in the specimens examined is a structure which does not normally exist, and must therefore have been formed during the enlargement of the organ. The capsule is in fact an integral part of the gland, as is shown by the presence within its layers of normal prostatic tissue. What happens would seem to be that as the adenomata enlarge, the surrounding tissue, whether it be normal prostate or that which has undergone glandular hyperplasia, is stretched over the more rapidly growing part and at the same time compressed, the result being that the glandular elements become less obvious, and the other elements become disposed in a laminated manner. Thus the "capsule" consists of the stretched and laminated prostatic tissue. The outcome of the whole investigation, which we may add appears to have been conducted in a most thorough manner, is that "there are no appearances presented by tumours removed from the prostate, and supposed to represent the whole organ, that cannot be accounted for equally well on the supposition that they are adenomata," and that the facts "seem to leave no reasonable doubt that the so-called total prostatectomy is nothing more than the removal of adenomatous masses."

[1] Brit. Med. Journ., March 29.

